# The real experience of patients after liver transplantation in intensive care unit: Retraction

**DOI:** 10.1097/MD.0000000000027322

**Published:** 2021-09-24

“The real experience of patients after liver transplantation in intensive care unit”,^[1]^ which appeared in Volume 100, Issue 29 of *Medicine* is being retracted. The authors contacted the Editorial Office requesting changes to the statistical sample after it was determined by the authors’ institution that the authors did not seek proper approval to use data from nine other institutions. As a result, the statistical sample changed from 320 to 8 patients, and greatly affected the statistical analysis and the results of the study.
